# Improved diagnosis of active *Schistosoma* infection in travellers and migrants using the ultra-sensitive in-house lateral flow test for detection of circulating anodic antigen (CAA) in serum

**DOI:** 10.1007/s10096-018-3303-x

**Published:** 2018-07-04

**Authors:** Rebecca van Grootveld, Govert J. van Dam, Claudia de Dood, Jutte J. C. de Vries, Leo G. Visser, Paul L. A. M. Corstjens, Lisette van Lieshout

**Affiliations:** 1 Department of Parasitology, Leiden University Medical Center, L4-Q, PO Box 9600, 2300 RC Leiden, The Netherlands; 2 Department of Medical Microbiology, Leiden University Medical Center, Leiden, The Netherlands; 3 Department of Molecular Cell Biology, Leiden University Medical Center, Leiden, The Netherlands; 4 Department of Infectious Diseases, Leiden University Medical Center, Leiden, The Netherlands

**Keywords:** Schistosomiasis, Diagnosis, Imported infections, Circulating anodic antigen (CAA), Serum, Travel medicine

## Abstract

Schistosomiasis is a parasitic disease affecting over 250 million people in the tropics. In non-endemic regions, imported *Schistosoma* infections are commonly diagnosed by serology, but based on antibody detection an active infection cannot be distinguished from a cured infection and it may take more than 8 weeks after exposure before seroconversion occurs. In endemic populations, excellent results have been described in diagnosing low-grade active *Schistosoma* infections by the detection of the adult worm-derived circulating anodic antigen (CAA) utilising robust lateral flow (LF) assays combined with up-converting phosphor (UCP) reporter technology. The purpose of this study is to explore the diagnostic value of the UCP-LF CAA assay in a non-endemic setting. CAA concentrations were determined in 111 serum samples originating from 81 serology-positive individuals. In nine individuals, serum could be collected before travel and an additional five provided samples before and after seroconversion occurred. Based on detectable CAA levels, an active infection was seen in 56/81 (69%) of the exposed individuals, while the 10 controls and the 9 sera collected before travel were tested negative for CAA. Positive CAA levels were observed starting 4 weeks after exposure and in four cases CAA was detected even before *Schistosoma*-specific antibodies became positive. Higher serum CAA levels were seen in migrants than in travellers and CAA concentrations dropped sharply when testing follow-up samples after treatment. This explorative study indicates the UCP-LF CAA serum assay to be a highly accurate test for detecting active low-grade *Schistosoma* infections in a non-endemic routine diagnostic setting.

## Introduction

Schistosomiasis is an infectious tropical disease caused by trematode blood flukes of the genus *Schistosoma*, affecting around 258 million people worldwide [1]. In the Netherlands, as in other Northwest European countries, schistosomiasis is an imported disease seen only in those who have been exposed in endemic regions, that is to say travellers originating from non-endemic countries who visited an endemic area or migrants who were born and raised there [2].

The classic method of detecting an infection with *Schistosoma* is microscopic examination of urine or faeces in the search for parasite eggs [3, 4]. When performed by well-trained and experienced technicians, microscopy is highly specific. But when the worm burden is low, as mostly seen in imported infections, this procedure generally lacks sensitivity [3–6].

The detection of specific antibodies against *Schistosoma* antigens is the most commonly applied alternative diagnostic approach in non-endemic routine diagnostic laboratories, especially for travellers who have been exposed for the first time [3, 5]. In general, antibody tests have good sensitivity with seroconversion mostly occurring 4 to 8 weeks after exposure, although some cases of late antibody detection have been described [5, 7, 8]. The major disadvantage of serology is that it does not distinguish between active and cured infection and the antibody levels do not give any information about the parasitic load [3, 5].

Detection of *Schistosoma* DNA in clinical samples is increasingly used in population-based surveys as a highly specific and more sensitive diagnostic alternative to microscopy and an increasing number of specialised research centres have implemented a *Schistosoma* DNA amplification method (i.e. real-time PCR) in their diagnostic package [9–11]. Parasite-specific DNA present in stool or urine samples presumably originates from the deposited eggs, which explains the generally observed correlation between stool or urine egg counts and *Schistosoma* DNA loads [3, 11]. Despite being more sensitive than microscopy, the sensitivity of *Schistosoma* DNA detection in stool or urine PCR is generally considered not to be high enough to justify PCR as the first and only diagnostic test for screening travellers and migrants [12].

Another approach to identifying active infection is by detection of *Schistosoma* antigens which are excreted in the human circulatory system. The best studied antigens are the worm-derived carbohydrate antigens circulating cathodic antigen (CCA) and circulating anodic antigen (CAA) [5]. Currently, two different monoclonal antibody-based lateral flow (LF) tests to detect these antigens in clinical samples are well accepted: (i) the user-friendly point of care strip assay for the detection of CCA in urine (POC-CCA) and (ii) the highly sensitive LF assay format for the detection of CAA in urine or blood derived samples (serum, plasma or dried blood stains) utilising fluorescent up-converting phosphor particles, the UCP-LF CAA assay [13–15]. The POC-CCA urine strip assay was developed according to ASSURED criteria for application in *Schistosoma mansoni*-endemic settings and is now generally accepted as an affordable and field-friendly semi-quantitative assay for prevalence mapping of this species [16–18]. The UCP-LF CAA assay is a genus-specific test, detecting all known *Schistosoma* species including veterinary species. The UCP-LF CAA assay includes a sample preparation step and therefore requires some basic laboratory equipment. Moreover, the ultra-sensitive format of the test also includes a concentration step which adds to its sensitivity by allowing analysis of increased sample volume (e.g. 500-μL serum) [14, 15]. Resulting test strips are analysed with an adapted strip reader and the determined CAA concentrations allow for a more standardised output than the visual reading of the POC-CCA urine strips.

Several studies have shown that the UCP-LF CAA assay can effectively be used as a quantitative test to estimate *Schistosoma* worm burdens at genus level in endemic populations, even when egg counts are very low [19–21]. Therefore, the UCP-LF CAA test might be specifically helpful in diagnosing schistosomiasis in travellers and migrants. In this explorative study, we evaluate whether the detection of CAA in serum has potential diagnostic value within a routine diagnostic laboratory in a non-endemic setting.

## Materials and methods

### Clinical samples

Serum CAA concentrations were measured in 121 serum samples from in total 91 individuals originating from two different studies (Table 1). The selection of the samples was based on the detectability of specific antibodies as part of routine diagnostic procedures. In short, *Schistosoma*-specific IgM antibodies directed against adult worm gut antigens were detected in an immunofluorescence assay (IFA) using sections of Rossmann’s fixed adult male *S. mansoni* worms, while IgG antibodies to *Schistosoma-*soluble egg antigens were detected in an enzyme-linked immunosorbent assay (ELISA) [5, 12]. All samples were anonymised before CAA testing.

**Table 1:** Classification of tested serum samples

Patient sera	Description	Number of individuals	Number of samples
Study A	Prospective		
Travellers	Non-endemic country of residence and pre- and 12 weeks post-travel sampling	9	18
Study B	Retrospective		
Travellers	Non-endemic country of residence and exposition less than 6 months ago and positive serology (at least IFA-positive)	27	34
Migrants	Immigrants or expatriates from a *Schistosoma*-endemic region, suspected for a chronic *Schistosoma* infection and positive serology (at least IFA-positive)	32	36
	Above and proven active infection (microscopy and/or PCR-positive)	10	14
Undefined	Exposed, but unable to categorise into either travellers or migrants due to deficient clinical information and positive serology (at least IFA-positive)	3	9
Negative controls	Submitted for *Schistosoma* and *Strongyloides* serology although clinically not highly suspected and negative serology (IFA and ELISA)	10	10
Total		91	121

The first (A) of the two studies includes 18 samples originating from a prospective cohort study in which 146 asymptomatic long-term travellers were tested for *Schistosoma*-specific antibodies before and 3 months after returning from their travel to Sub-Saharan Africa [12]. Nine travellers were found to be seroconverted of which one also had detectable *Schistosoma* DNA in stool, confirming an active infection [11, 12]. Serum CAA concentrations were determined in each of the nine individuals before travel and after their return. All were treated with praziquantel, but no further follow-up samples were collected [12].

The second (B) of the two studies includes 103 samples which were retrospectively selected from a stored collection of serum samples submitted to our laboratory because of a suspected *Schistosoma* infection. Based on the limited recorded information, 27 of the 82 selected individuals could be categorised as antibody-positive travellers known to be exposed in the previous months, while 42 individuals could be described as antibody-positive migrants with a suspected chronic *Schistosoma* infection. Ten of these migrants showed signs of active *Schistosoma* infection, i.e. detectable eggs in stool or urine or a positive outcome in the *Schistosoma* PCR. Three additional individuals of study B could not be identified as being either traveller or migrant, but were chosen based on the fact that consecutive samples were submitted, of which at least one sample showed detectable antibodies. Finally, ten subjects were included as negative controls based on negative serology and showing no other indications of schistosomiasis (Table 1).

### UCP-LF CAA assay

Serum samples were stored at − 20 °C until tested by the UCP-LF CAA assay (SCAA500) as described before [15]. In brief, 500-μL serum was mixed with an equal amount of 4% (*w*/*v*) trichloro-acetic acid (TCA) in order to remove interfering proteins and to dissociate potential immune complexes. Following a centrifugation step, 500-μL supernatant was transferred into an Amicon Ultra-0.5 Centrifugal Filter Device (Merck Millipore) and concentrated to approximately 20 μL. All concentrated samples were examined by the wet-format assay, which includes a sonication step of the UCP stock solution, while 28 samples were additionally tested by the more user-friendly dry-format assay [15, 22]. According to previous protocols, serum sample concentrates were mixed with CAA-specific UCP reporter conjugate solution and incubated for 1 h on a microtiter plate thermo-shaker (37 °C, 900 rpm), after which a LF strip was placed in each well [14]. The UCP signal test (T) line on the strip contains the same mouse monoclonal anti-CAA antibody (#147-3G4, Dept. of Parasitology, Leiden University Medical Center (LUMC)) as the UCP reporter conjugate, while the flow control (Fc) line contains an anti-mouse antibody. The strips were scanned for UCP reporter signals with a dedicated Packard FluoroCount strip reader [22]. Results were expressed as a ratio value between the T line and the Fc line. A TCA-soluble fraction of *S. mansoni* adult worm antigen with known CAA concentration was used as a reference standard for the quantification of the antigen [15].

The lower limit of detection of the UCP-LF CAA assay is known to show some batch-to-batch variation, but should be at least be 0.5 and 1.5 pg CAA/mL for the SCAA500 wet format and dry format, respectively [15]. Here, the available batch of assay materials allowed a lower limit of detection of 0.1 and 0.6 pg CAA/mL, respectively.

### Data analysis

CAA concentrations and antibody titres were arbitrarily categorised into different intensity groups as indicated in Table 2. The Spearman correlation test was used to analyse the relationship between antibody levels and CAA concentrations and the relationship between the output of the wet-format and dry-format assay.

**Table 2:** Anti-*Schistosoma* antibody titres and serum circulating anodic antigen (CAA) concentration determined by the wet-format 500-μL UCP-LF CAA assay

	Prospective study A		Retrospective study B^a^		
	Before travel *n* = 9 (%)	After travel *n* = 9 (%)	Controls *n* = 10 (%)	Travellers *n* = 27 (%)	Migrants *n* = 42 (%)
IFA: Ab against worm antigens^b^					
High	–	6 (67)	–	16 (59)	7 (17)
Moderate	–	3 (33)	–	11 (41)	25 (60)
Low	–	–	–	–	10 (24)
Negative	9 (100)	–	10 (100)	–	–
ELISA: Ab against egg antigens^c^					
High	–	–	–	1 (4)	8 (19)
Moderate	–	2 (22)	–	9 (33)	28 (67)
Low	–	–	–	4 (15)	5 (12)
Negative	9 (100)	7 (78)	10 (100)	13 (48)	1 (2)
CAA (pg/mL)^d^					
High	–	–	–	–	8 (19)
Moderate	–	–	–	2 (7)	9 (21)
Low	–	3 (33)	–	5 (19)	8 (19)
Marginal	–	2 (22)	–	8 (30)	8 (19)
Negative	9 (100)	4 (45)	10 (100)	12 (44)	9 (22)

^a^Undefined subjects were excluded

^b^IFA antibody titres were arbitrarily categorised into different groups: high ≥ 1:512; moderate 1:64–1:256; low 1:16–1:32; negative < 1:16

^c^ELISA antibody titres were arbitrarily categorised into different groups: high ≥ 1:1024; moderate 1:128–1:512; low 1:32–1:64; negative < 1:32

^d^CAA concentrations (pg/mL) were categorised into different groups: high > 100; moderate 10–100; low 1–10; marginal 0.1–1; negative < 0.1

## Results

Table 2 summarises the detected *Schistosoma* antibody titres and serum CAA concentrations as determined by the wet-format SCAA500 assay. In those cases of the retrospective study (B) where multiple samples per individual were included, the findings of the last sample collected before drug treatment are depicted.

In the prospective study (A), all nine were negative by definition in the *Schistosoma* serology before travelling. After their return, all travellers showed moderate to high antibody titres against the worm antigens in the IFA, while only two (22%) showed antibodies against egg antigens. Before travel, all were CAA-negative in serum, while 5/9 (56%) had marginal to low CAA levels after exposure, ranging from 0.5 to 2.7 pg/mL. Among the five CAA-positive individuals were the two travellers with anti-egg antibodies and the one with detectable *Schistosoma* DNA in stool.

In the retrospective study (B), travellers showed higher antibody titres against worm antigens than migrants, while on the other hand, migrants showed higher antibody titres against egg antigens. Anti-egg antibodies could be detected in 98% of the 42 migrants, compared to 52% of the 27 travellers (Table 2). The single migrant being anti-egg antibody-negative was a patient with PCR-proven chronic schistosomiasis. By definition, the ten negative controls showed no specific antibodies in the IFA or ELISA. No serum CAA could be demonstrated in any of these negative controls, while detectable CAA levels were found in 33/42 (79%) of the migrants and 15/27 (56%) of the travellers. Antigen levels were found to be higher in the migrants, with 8/42 (19%) showing a CAA concentration of at least 100 pg/mL, compared to none of the travellers (Table 2). The three individuals without known travel history were all CAA-positive with levels ranging from 0.1 to 1.9 pg/mL. All ten individuals of study B with a proven active infection, i.e. detectable eggs and/or *Schistosoma* DNA in stool or urine, were positive for CAA. No correlation was seen between serum CAA levels and antibody titres.

**Fig. 1:**
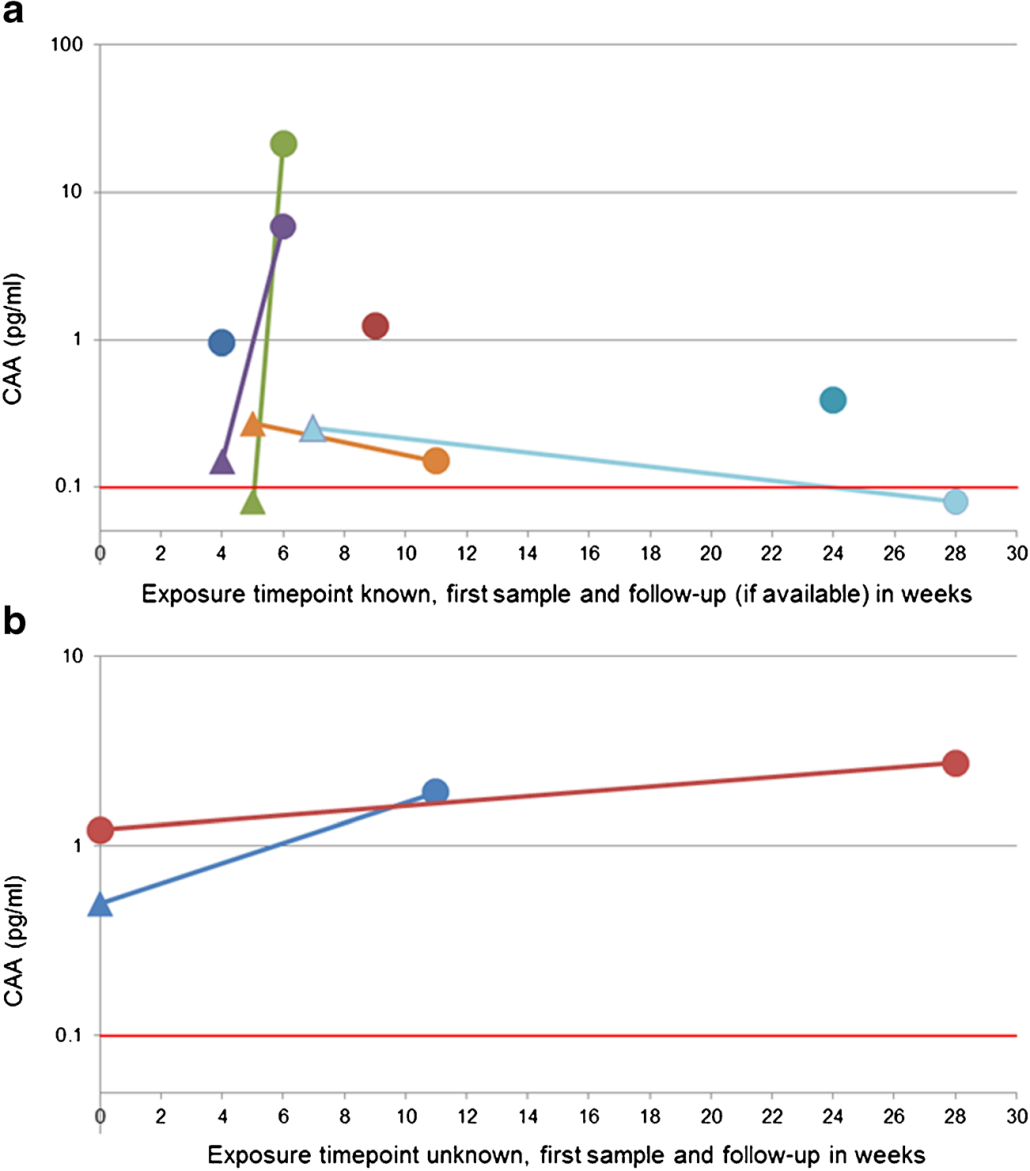
**a**, **b** Serum CAA concentration after known date of exposure (**a**) and unknown date of exposure (**b**) of subjects in retrospective study B. Triangle indicates before seroconversion, i.e. IFA and ELISA still negative. Circle indicates after seroconversion. Red line indicates 0.1 pg CAA/mL and UCP-LF CAA assay threshold value

**Fig. 2:**
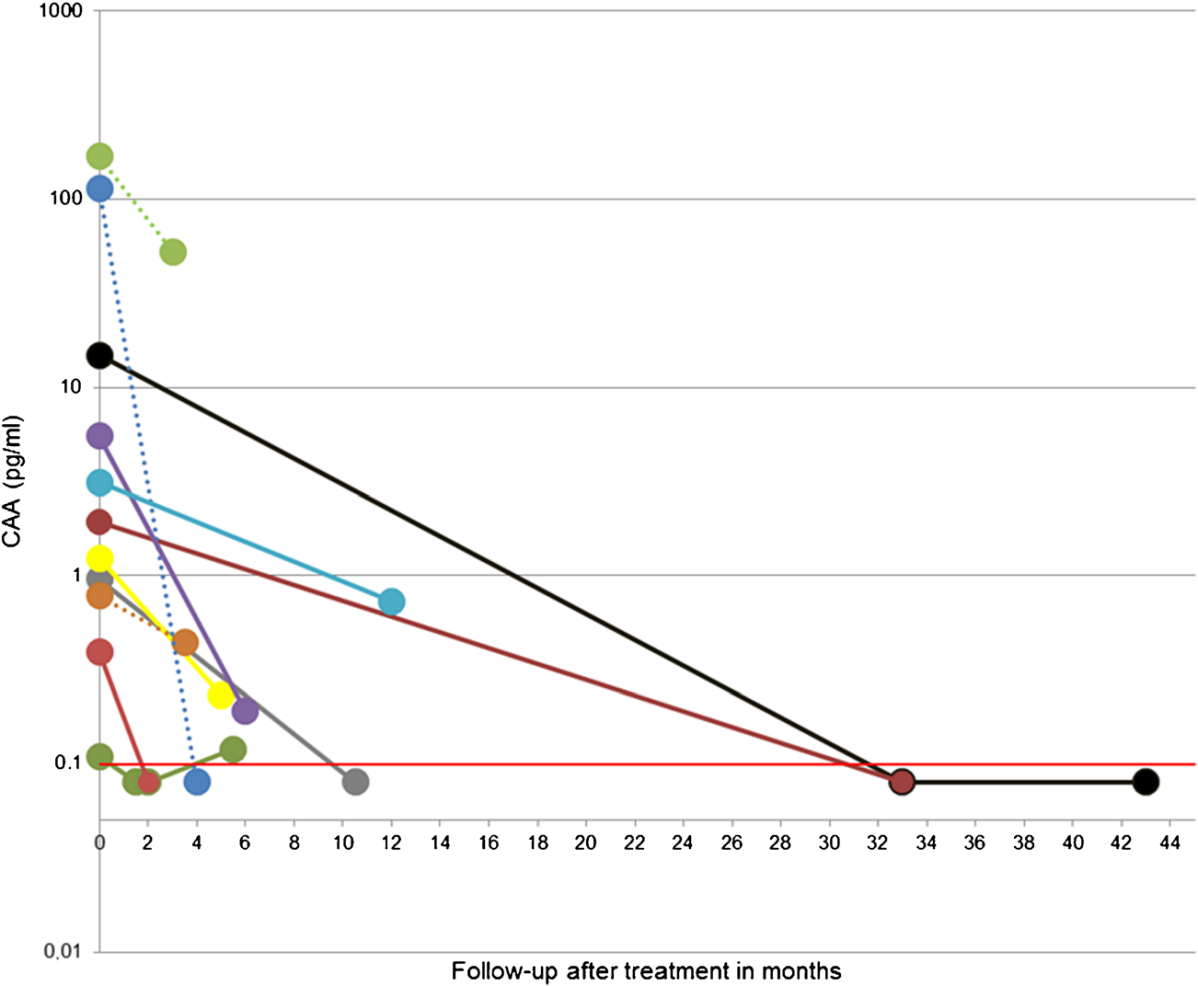
Serum CAA concentration after treatment of subjects in retrospective study B. Dotted line indicates treatment very likely but not formally recorded. Red line indicates 0.1 pg CAA/mL and UCP-LF CAA assay threshold value

In study B, the approximate date of exposure was known for seven travellers (Fig. 1a) and two additional individuals had a second serum sample tested before treatment was applied (Fig. 1b). In the four individuals, detectable CAA was found in the sample previous to the sample being antibody-positive (Fig. 1) and the earliest detection of CAA was 4 weeks after exposure (Fig. 1a). The outcome of post-treatment follow-up samples is shown in Fig. 2. All 11 examined individuals, a mixture of travellers and migrants, showed a decline in CAA concentration, although one increased again when tested in a follow-up 2 weeks later.

**Fig. 3:**
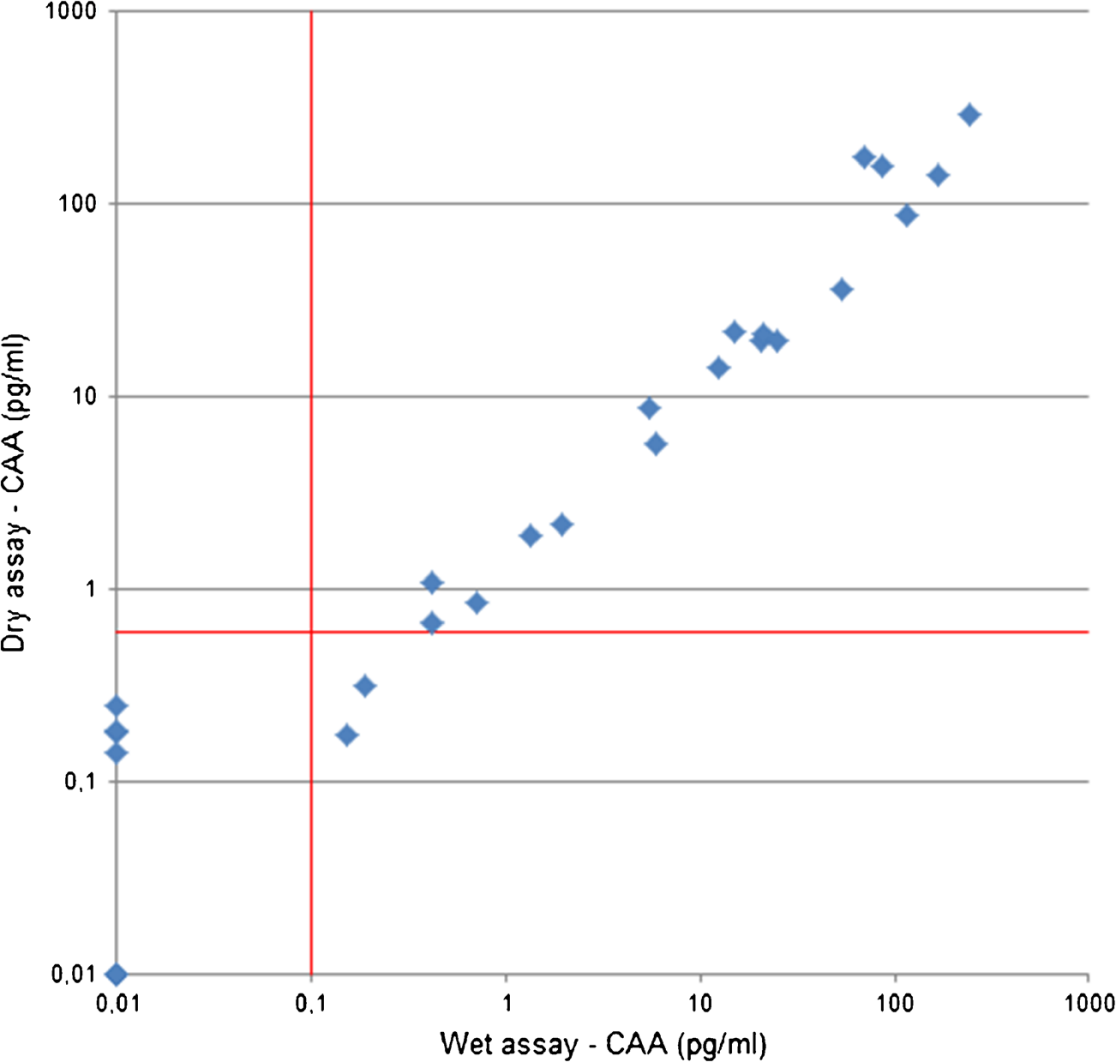
Serum CAA concentration of samples performed in the dry-format and wet-format SCAA500 assay Red lines indicate the UCP-LF CAA assay threshold value, dry-format assay 0.6 pg CAA/mL and wet-format assay 0.1 pg CAA/mL. Correlation in CAA concentrations determined by the two tests: Spearman’s rho = 0.97, *n* = 28, *P* < 0.001

A sub-group of 28 CAA-positive sera were additionally tested by the more user-friendly dry-format assay. CAA concentrations determined by the two tests correlated significantly, but two samples tested CAA-negative while marginal CAA levels were detected by the wet-format assay (Fig. 3).

## Discussion

In this study, we further explored the ultra-sensitive UCP-LF assay for the detection of *Schistosoma* circulating antigen CAA in serum of travellers and migrants, a patient group known to harbour mostly low worm burdens. The number of individuals with detectable CAA levels in serum ranged from 55% in antibody-positive travellers to 78% in antibody-positive migrants and CAA could be detected within weeks after exposure. All individuals with proven active infection were CAA-positive and a sharp decrease in serum CAA concentrations was observed following anti-schistosomal treatment.

Diagnosing schistosomiasis via the detection of adult worm-related schistosomal circulating antigens has been employed extensively within populations living in endemic areas, but limited data is available on the detection of CAA in imported *Schistosoma* infections [19–21]. Because of its ultra-sensitive features and its capacity to diagnose all *Schistosoma* species, the UCP-LF CAA assay has much higher potential than the POC-CCA urine strip to be successfully implemented as a routine diagnostic laboratory test for the diagnosis of schistosomiasis in travellers and migrants [23, 24].

In the current study, CAA could be detected starting 4 weeks after exposure and in four cases CAA was demonstrated even before *Schistosoma*-specific antibodies became positive. In a previous study, CAA could be detected in a cohort of travellers within approximately 6 weeks after exposure using a relatively less sensitive ELISA format [25]. Studies in animal models have shown that both CAA and CCA can be found even earlier, but in these models, infection levels were mostly high [15, 26].

Surprisingly, we found only five of the nine asymptomatic travellers to have detectable serum CAA levels 3 months after their return from Sub-Saharan Africa. A possible explanation of missing CAA in the remaining seroconverted travellers could be, besides an extremely low worm burden, that the infection initially started after exposure, but aborted spontaneously over time, leaving only specific anti-adult worm antibodies to remain. None of these CAA-negative individuals showed antibodies against soluble egg antigens.

A limitation of the current study is its mostly retrospective design. The LUMC functions as a reference centre for antibody-based *Schistosoma* diagnostics within the Netherlands. For the majority of the submitted serum samples, detailed clinical information is lacking. Therefore, it cannot be excluded that some of the included patients have been treated for schistosomiasis, either on a presumptive base or because of the outcome of microscopy by the local hospital. For example, one traveller was CAA-positive when tested 7 weeks after returning from a *Schistosoma-*endemic region, but tested negative for specific antibodies. At 28 weeks after exposure, a new serum sample was submitted and this time, the sample tested positive in serology, but CAA was no longer detectable (Fig. 1a). Possibly, this patient has been treated with praziquantel after the first sample was collected.

In the present study, the user-friendly dry-format assay was evaluated for the first time utilising the SCAA500 assay on clinically relevant samples in a non-endemic setting. Importantly, this format overcomes a sonication step, thereby simplifying the procedure substantially [22]. Detected CAA concentrations were highly comparable and although being slightly less sensitive, this assay format is an important next step towards the implementation of the UCP-LF CAA assay as a routine diagnostic procedure.

In conclusion, we found the serum UCP-LF CAA assay to be a useful tool for the diagnosis of an active *Schistosoma* infection in a non-endemic setting. For implementation of this assay in routine diagnostic laboratories, logistical aspects such as ease of use, robustness and reproducibility are just as important as high-test specificity and sensitivity. At the moment, the UCP-LF CAA assay is only available as a research tool and some technical aspects of the assay protocol may need adaptation before it will be officially approved as a (commercial) diagnostic test. Nonetheless, current data demonstrate the suitability of the UCP-LF CAA assay for diagnosing low parasite burdens in migrants and travellers.
